# Web-based climate information resources for malaria control in Africa

**DOI:** 10.1186/1475-2875-5-38

**Published:** 2006-05-11

**Authors:** Emily K Grover-Kopec, M Benno Blumenthal, Pietro Ceccato, Tufa Dinku, Judy A Omumbo, Stephen J Connor

**Affiliations:** 1 International Research Institute for Climate and Society, The Earth Institute at Columbia University, Lamont Campus, 61 Route 9W, Palisades, New York 10964, USA

## Abstract

Malaria remains a major public health threat to more than 600 million Africans and its control is recognized as critical to achieving the Millennium Development Goals. The greatest burden of malaria in Africa occurs in the endemic regions where the disease pathogen is continuously present in the community. These regions are characterized by an environment that is conducive to interactions between the Anopheles mosquito, malaria parasites and human hosts, as well as housing of generally poor quality, which offers little protection from mosquito-human contact. Epidemic malaria tends to occur along the geographical margins of endemic regions, when the equilibrium between the human, parasite and mosquito vector populations is occasionally disturbed and a sharp but temporary increase in disease incidence results. When malaria control measures are inadequate, as is the case in much of sub-Saharan Africa, the disease distribution is closely linked with seasonal patterns of the climate and local environment. In the absence of good epidemiological data on malaria distribution in Africa, climate information has long been used to develop malaria risk maps that illustrate the boundaries of 'climatic suitability for endemic transmission.' The best known of these are produced by the Pan-African-based MARA Collaboration. This paper describes the development of additional malaria suitability maps which have been produced in an online, interactive format to enable temporal information (i.e., seasonality of climate conditions) to be queried and displayed along with spatial information. These maps and the seasonal information that they contain should be useful to the malaria control and health service communities for their planning and operational activities.

## Introduction

Malaria remains a major public health threat to the African continent and its control is critical to achieving the Millennium Development Goals in this region. The recently published Global Strategic Plan for Roll Back Malaria 2005–2015 has stated that "Six out of eight Millennium Development Goals can only be reached with effective malaria control in place" [1]. The greatest burden of malaria in Africa is born by populations in regions where the disease pathogen is perennially present in the community. In these regions, the environment is conducive to interactions between the Anopheles mosquito, malaria parasites and human hosts because they contain surface water in which mosquitoes can lay their eggs, humid conditions which facilitate adult mosquito life spans of adequate length, and relative warmth which allows both the mosquito and the malaria parasite to develop rapidly. In addition, housing quality is generally poor and offers little protection from human-mosquito interaction. Those most vulnerable to endemic malaria are young children (<5 years of age) who have yet to acquire disease immunity, pregnant women, whose immunity is reduced, and non-immune migrants or travelers.

Epidemic malaria tends to occur along the geographical margins of the endemic regions, when the conditions supporting the equilibrium between the human, parasite and mosquito vector populations are disturbed. This leads to a sharp but temporary increase in disease incidence. More than 124 million Africans live in such areas and experience epidemics causing around 12 million malaria episodes and up to 310,000 deaths annually [2]. In these regions, an individual's exposure to malaria is infrequent and, therefore, little acquired immunity to this life threatening disease is developed. All age groups are, therefore, vulnerable to epidemic malaria [3]. The development of an online product that supports epidemic risk monitoring has been previously reported [4].

While economic development has played an enormous role in shaping the current distribution of endemic malaria, the seasonal characteristics of climate have a significant relationship with the distribution and seasonality of the disease where control efforts have been largely unsuccessful. Specifically, malaria may be found where and when the climatic conditions are favorable for transmission between the mosquito vector and its human host. It then follows that understanding and monitoring these climate conditions and their relationship with malaria incidence is of vital importance to limiting the impacts of the disease.

### Risk maps and seasonal calendars for endemic malaria control planning

In the absence of good epidemiological data on malaria distribution in Africa, climate information has long been used to develop malaria risk maps that illustrate the boundaries of 'climatic suitability for endemic transmission'. Such maps date back at least one hundred years [5]. The most widely cited of these have been developed in recent years by the Pan-African-based MARA Collaboration [6,7]. New malaria suitability maps, which have been produced in an online interactive format to enable graphic temporal information (i.e., seasonality of suitable climate conditions) to be queried and displayed along with the spatial information (i.e., spatial extent of suitable climate conditions), are described below [8]. Such seasonal information should help with the development of malaria control calendars and assist health services in appropriately focusing their control activities, such as drug procurement and anti-vector spraying. Seasonal calendars may also help avoid misdiagnosis and inappropriate drug treatment, which are vital to reducing the development rate of parasite-drug resistance in Africa.

### The role of climate

Rainfall is largely responsible for creating the conditions which allow sufficient surface water for mosquito breeding sites and is, therefore, recognized as one of the major factors influencing malaria transmission. Temperature also plays an important role in the variability of malaria transmission by regulating the development rate of mosquito larvae and influencing the survival rate of adult mosquitoes. Mosquitoes generally develop faster and feed earlier in their life cycle and at a higher frequency in warmer conditions. In addition, the Plasmodium parasite multiplies more rapidly in the mosquito in higher temperatures [9,10]. Humidity impacts the survival rate of the mosquito as well. Mosquitoes will generally not live long enough to complete their transmission cycle where and when the relative humidity is consistantly less than 60% [9,11]. In endemic regions these three variables usually create conditions suitable for malaria transmission every year. In the epidemic prone regions one of these variables is typically not sufficient to support transmission.

## Discussion

Staff at the International Research Institute for Climate and Society (IRI) have recently created an online resource for information about the climate conditions that are associated with malaria transmission. The 'Climate and Malaria Resource Room' contains background products illustrating the historical and modeled occurrence of climate conditions that are suitable for malaria transmission in Africa as well as dynamic products that monitor some of these conditions (i.e. rainfall) for epidemic early warning purposes [4]. These monitoring products are automatically updated when new data become available. The Climate and Malaria Resource Room resides in the IRI Data Library Map Room [12]. The maps and the data on which they are based are freely available to the public and can be downloaded in a variety of formats, including four GIS-compatible options. A brief description of the maps and interfaces that are currently available in the Resource Room, including updates to an existing epidemic monitoring product [4], is also provided below.

### Seasonal climatic suitability for malaria transmission (CSMT)

**Figure 1:**
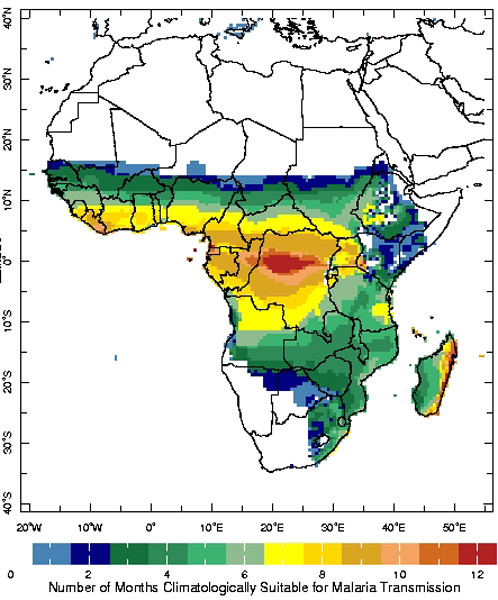
Clickable map used in the Seasonal Climatic Suitability for Malaria Transmission interface illustrating the number of months during the year that are suitable for malaria transmission based on climatological conditions.

The CSMT tool is an interactive map that displays the number of months during the year when climatological (i.e., long-term average) conditions are considered to be suitable for malaria transmission (Figure 1). Suitability is based on empirically-derived thresholds of precipitation, temperature and relative humidity [13,14]. For the purposes of this tool, climatic conditions are considered to be suitable for transmission when the monthly precipitation accumulation is at least 80 mm, the monthly mean temperature is between 18°C and 32°C and the monthly relative humidity is at least 60% [6,9]. These thresholds are based on a consensus of the literature. In practice, the optimal and limiting conditions for transmission are dependent on the particular species of the parasite and vector.

**Figure 2:**
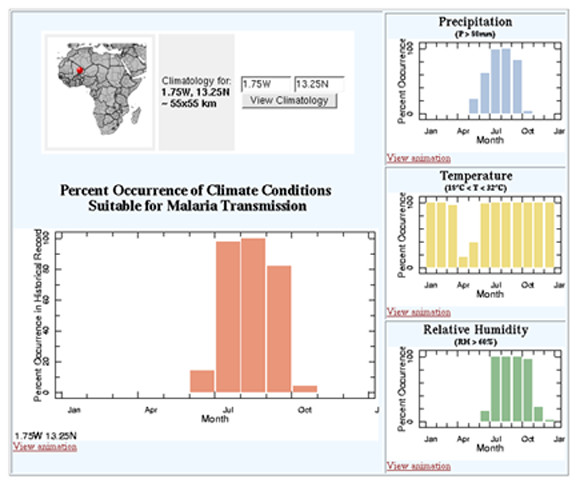
Time series graphs displaying the percent occurrence of climate conditions that were suitable for malaria transmission during 1951–2000 at the location of interest. Graphs for central Burkina Faso are shown.

**Figure 3:**
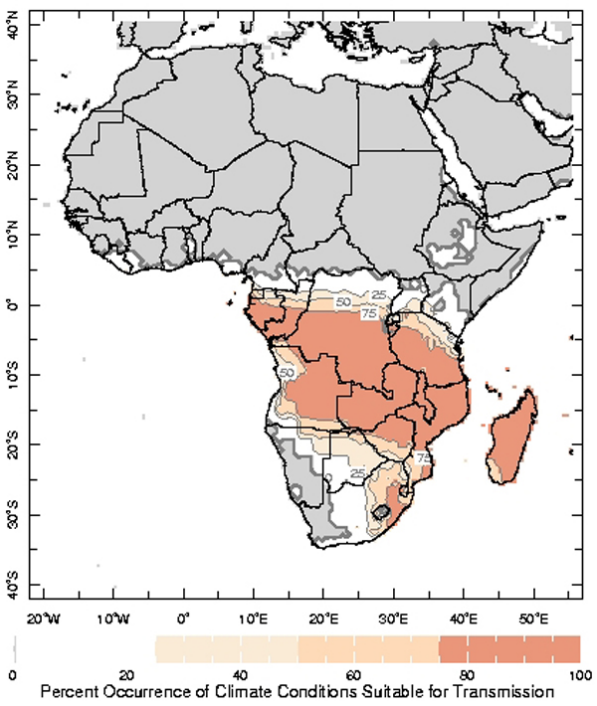
A single frame (January) of the 12-month animation of the percent occurrence of climate conditions which are suitable for malaria transmission during 1951–2000.

Users may gain insight into how often these conditions have actually occurred during any particular time of the year by clicking on the map at the location of interest. The resulting page illustrates the frequency of occurrence of the precipitation, temperature and relative humidity conditions, as well as that for all three conditions occurring coincidentally, expressed as the percent occurrence during the 50-year reference base period (i.e., 1951–2000; Figure 2). Animated images of the percent occurrence of each of these conditions across the African continent are also available (Figure 3).

These figures, and the empirically-derived thresholds on which they are based, provide a macro-scale illustration of where and when climatically-suitable conditions for malaria transmission exist. It should be noted, however, that mosquitoes will actively seek out micro-climatic conditions that maximize their chances of survival. This, along with other local characteristics, such as the presence of water containers, will have a significant bearing on actual transmission.

### MARA model of climatic suitability for malaria transmission

The distribution model of climatic suitability for malaria transmission developed by Craig *et al*. [6] is also included in the Climate and Malaria Resource Room. Like the CSMT, it is not based on actual malaria data, but this fuzzy-logic model is based on long-term climatological averages and, therefore, provides insight into the potential for transmission across the continent during the average year. More information about the distribution model and related products is available at the MARA website [15].

### Malaria Early Warning System (MEWS)

The MEWS interface has been in the public domain since November 2004 and supports the understanding of the current rainy season by providing a seasonal and short-term historical context [4]. It displays the most recent dekadal rainfall estimates and allows users to generate four time-series graphs which provide an analysis of recent rainfall with respect to that of recent seasons and a short-term historical average.

This short-term historical average has replaced the long-term (i.e., 30-year) climatological reference utilized in the original release of the MEWS interface in order to provide a comparison of similar rainfall products. The dekadal rainfall data, for example, are satellite estimates that are adjusted with sampled surface rain gauges [16,17], while the original 30-year climatology was based on interpolated rain gauge data [13]. The inherent differences between these two datasets, and the underestimation of rainfall by the dekadal satellite monitoring product in particular, may have exaggerated negative anomalies while underestimating some positive anomalies. Users must note that the new short-term reference, which includes nearly all of the satellite-estimated data that is available (i.e., 6 years), should not be interpreted as a climatological normal, which is typically based on a much longer time period (i.e., 30 years). Despite the limitations that the short-term average imposes, the resulting precipitation analyses are still valuable in epidemic prone regions where the climate of the recent few years often has an important bearing on changes in community vulnerability and risk.

### Rainfall estimates compared to the short-term average

**Figure 4:**
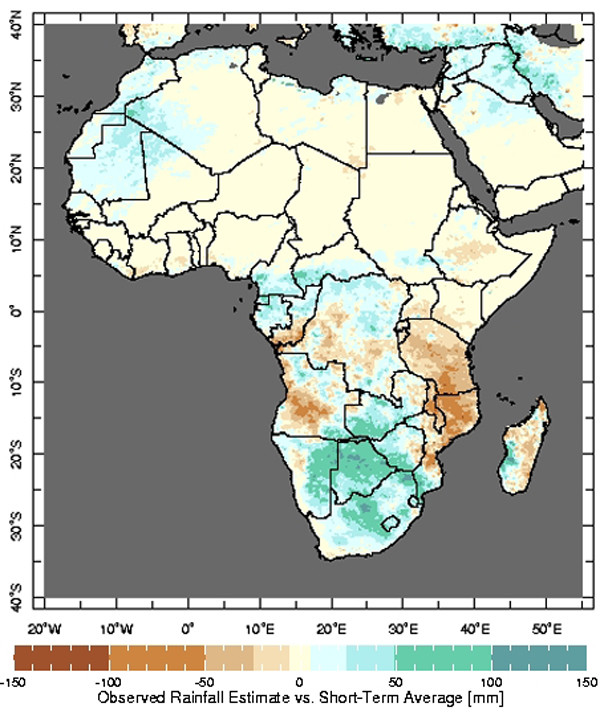
The Rainfall Estimate Differences map illustrating the difference between the estimated rainfall from the most recent dekad and the short-term average rainfall during the same time of the year.

**Figure 5:**
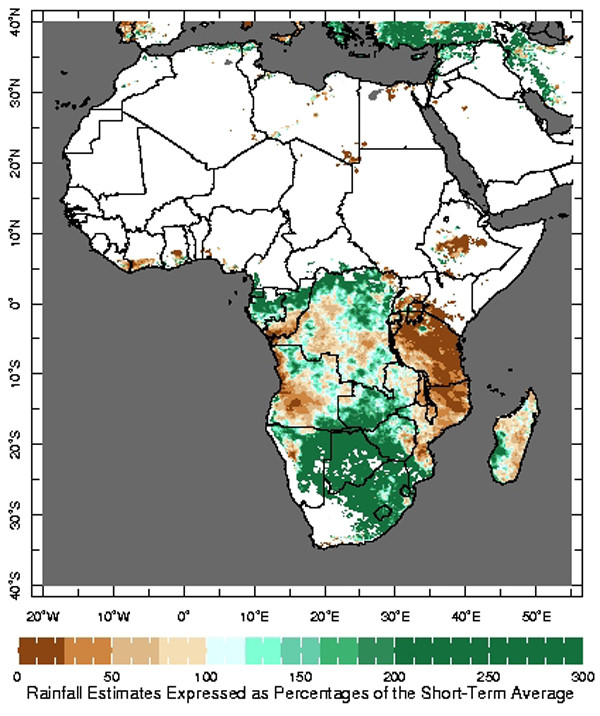
The Rainfall Estimate Percentages map illustrating the most recent dekadal rainfall estimates expressed as a percentage of the short-term average rainfall during the same time of the year. Only areas receiving at least 7 mm of rainfall per dekad, based on the short-term average, are included in the analysis.

Users of the MEWS interface, particularly those involved in malaria control, have indicated that it is beneficial for them to have access to climate information that is presented in a variety of ways. In response to that feedback, two additional maps that compare the most recent dekadal rainfall estimates to the short-term average have been developed. The Rainfall Estimate Differences map displays the difference between the most recent dekadal rainfall estimates and the same short-term historical average utilized in the MEWS interface (Figure 4). The second map, Rainfall Estimate Percentages, illustrates the most recent dekadal rainfall estimates as percentages of the short-term average (Figure 5). The same visual features (e.g., overlays, zoom) that are available in the MEWS interface are available on these maps as well.

### Summary and future work

While economic development has played an enormous role in shaping the current distribution of endemic malaria, the seasonal characteristics of climate have a significant relationship with the distribution and seasonality of the disease where it is not adequately controlled. A new interactive climate information tool has been developed to facilitate the understanding of the seasonal nature of climatic suitability for endemic transmission. This tool is part of a Climate and Malaria Resource Room, which also contains dynamic, automatically-updated products that monitor rainfall for epidemic early warning purposes. Future work will include the development of an interactive tool for disseminating vegetation data based on TERRA-MODIS images at 250 m spatial resolution (i.e., Normalized Difference Vegetation Index and single channels to create different indices useful to retrieve vegetation, bare soils and water bodies). A French language version of the Climate and Malaria Resource Room is also underdevelopment. The authors welcome the opportunity to work with other groups to incorporate their models and monitoring products into this online resource.
